# Genome-wide association identifies novel ROP risk loci in a multi-ethnic cohort

**DOI:** 10.21203/rs.3.rs-2855404/v1

**Published:** 2023-05-16

**Authors:** Jerome Rotter, Xiaohui Li, Leah A. Owen, Kent Taylor, Susan Ostmo, Yii-Der Ida Chen, Aaron Coyner, Kemal Sonmez, M.Elizabeth hartnett, Xiuqing Guo, Eli Ipp, Kathryn Roll, Pauline Genter, R.V. Paul Chan, Margaret DeAngelis, Michael Chiang, J Peter Campbell

**Affiliations:** Lundquist Institute; The Institute for Translational Genomics and Population Sciences; University of Utah, Moran Eye Center; The Institute for Translational Genomics and Population Sciences; Oregon Health & Science University; Lundquist Institute for Biomedical Innovation at Harbor-UCLA Medical Center; Oregon Health & Science University; Casey Eye Institute Oregon Health & Science University; University of Utah; The Institute for Translational Genomics and Population Sciences; THE LUNDQUIST INSTITUTE; THE LUNDQUIST INSTITUTE; THE LUNDQUIST INSTITUTE; University of Illinois at Chicago; SUNY-University at Buffalo; National Eye Institute; OHSU

## Abstract

We conducted a genome-wide association study (GWAS) in a multiethnic cohort of 920 at-risk infants for retinopathy of prematurity (ROP), a major cause of childhood blindness, identifying 2 loci at genome-wide significance level (p<5×10–8) and 7 at suggestive significance (p<5×10–6) for ROP ≥ stage 3. The most significant locus, rs2058019, reached genome-wide significance within the full multiethnic cohort (p=4.96×10–9); Hispanic and Caucasian infants driving the association. The lead single nucleotide polymorphism (SNP) falls in an intronic region within the Glioma-associated oncogene family zinc finger 3 (GLI3) gene. Relevance for GLI3 and other top-associated genes to human ocular disease was substantiated through in-silico extension analyses, genetic risk score analysis and expression profiling in human donor eye tissues. Thus, we report the largest ROP GWAS to date, identifying a novel locus at GLI3 with relevance to retinal biology supporting genetic susceptibilities for ROP risk with possible variability by race and ethnicity.

## Introduction

Retinopathy of prematurity (**ROP**) is a retinal vascular disease that affects premature infants and is a leading cause of childhood blindness worldwide^1–3^. Birth weight (**BW**) less than 1500 grams, prematurity less than 32 weeks gestational age (**GA**), and post-natal oxygen exposure confer independent ROP risk^4,5^. However, this understanding of risk and pathogenesis is incomplete as only approximately 50–65% of infants with these risk factors will develop ROP disease^4,6,7^. For example, phenotypic extremes that do not conform to current risk profiles exist; preterm infants born at large birth weights and/or older gestational ages may develop treatment-warranted ROP whereas those within the risk profiles of GA and BW do not always develop treatment-warranted ROP^8^. The current stratification also does not address prevention; although post-natal oxygen exposure is the most modifiable risk, limiting oxygen increases infant mortality^9–11^. As a result, current screening lacks specificity and interventions are unable to modify preclinical ROP risk, instead targeting ROP once retinal disease is present and there is significant risk of blindness^6,12^. Improved understanding of risk will allow better detection and treatment of infants with greatest risk for severe ROP and may provide novel insights into disease patho-mechanisms in order to prevent disease.

ROP risk is multifactorial and although genetic risk is not fully elucidated, there is a growing body of evidence supporting a genetic basis for ROP, including twin studies^13–15^. Case control and whole exome candidate approaches have identified significant risk associations between single nucleotide polymorphisms (SNP) in *brain-derived neurotrophic factor* (*BDNF*; rs7934165 and rs2049046), *thrombospondin type-1 domain-containing protein 4* (*THSD4*), *TNF* -308G/A polymorphism and *angiotensin 1 converting enzyme* insertion deletion (*ACE* ID) polymorphism^16–19^ (Supplementary Table 1). By contrast, recent candidate work demonstrated significant protective associations between the *BDNF* SNP rs7929344 and ROP and severe ROP risk associations between SNPs within *VEGFA*, *NOS3* and *EPAS1*^20^. While no SNP has been shown to reach genome-wide significance, meta-analysis has substantiated some associations^18^ while also beginning to identify pathobiology underlying observed differences in ROP risk relative to race; an example being recent work demonstrating association of the *VEGFA*+405G>C polymorphism association with ROP risk in Caucasian populations^21^. This observation aligns well with clinical observations showing racial differences in risk, including greater ROP severity and vision loss in white as compared to black preterm infants with equivalent BW and GA ROP risk^22,23^. Further, clinical progression has been shown to vary in Hispanic compared to white non-Hispanic populations^24^.

Taken together, our current understanding of ROP risk is incomplete, which impairs our ability to predict infants at greatest disease risk or identify pathobiology allowing for ROP prevention. While ROP risk is multifactorial, evidence supports genetic contributions to risk and protection. Herein we present a GWAS analysis based within the i-ROP consortium, including collaborators from 14 academic institutions throughout the world, demonstrating a novel ROP risk association with the SNP *GLI3 rs2058019* reaching genome-wide significance (p=4.90E-09) within our multi-ethnic cohort. We further demonstrate broader applicability for this SNP and *GLI3* genetic variation with other forms of retinal disease characterized by pre-retinal neovascularization, namely diabetic retinopathy. Finally, we identify additional SNPs with suggestive-level significance association with severe ROP disease, demonstrate cross-significance with previously identified ROP-associated SNPs, and further demonstrate relevance for GLI3 and these top SNPs within the human eye, finding expression in both human donor neurosensory retina as well as retinal pigment epithelium (RPE).

## Results

### iROP GWAS Multi-ethnic Cohort Characteristics:

The iROP cohort consists of 2187 preterm infants born at gestational ages less than 32 weeks and birth weights smaller than 1250 grams, placing them at risk to develop ROP. The iROP database was mined for infants with both biologic and phenotypic data for subsequent genotype-phenotype analysis. To identify genetic susceptibility regions for ROP, we conducted a genome-wide association study (GWAS) using the Illumina Infinium Global Screening Array with information from these infants. As published previously, phenotype was rigorously determined by a team of ophthalmologists and image graders with clinical expertise in ROP^16,25,26^. This subset of iROP patients represents multiple ethnic and racial groups, including 44.5% Hispanic, 35.4% White, 12.1% African American and 8% unidentified race individuals as noted in Table 1. Further, all ROP phenotypes are represented, including 197 with ROP Stages 3, 4, or 5. (Table 1). The distribution of ROP disease, ethnicity and race was similar between the infants with and without genetic data (Supplementary Table 2).

### A number of SNPs demonstrate significant associations with severe ROP.

**Figure 1:**
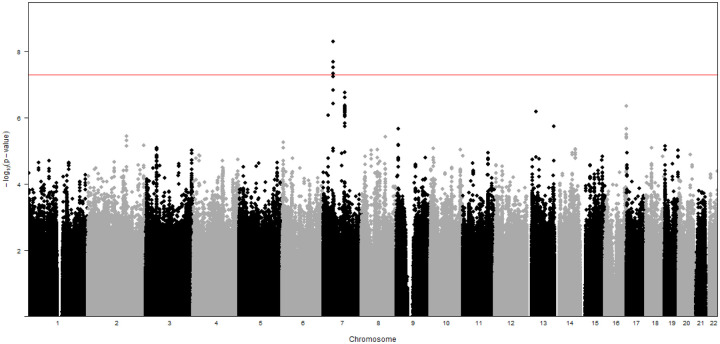
Manhattan plot of genome-wide association with ROP severity (stage 3 or greater): Data were analyzed for all populations combined. The red line on indicates genome-wide significance (p≤ 5×10^−8^)

GWAS analysis was performed on DNA extracted from all 920 cohort infants to identify genetic susceptibility regions for ROP severity, defined as ROP stage 3 or greater. As depicted in Figure 1, which visualizes all p values of SNP-associations for the 22 autosomal chromosomes plotted relative to chromosome location and significance, a number of SNP associations were identified. The Y axis represents the −log transformed p value (red line representing GWAS significance level) and X axis is in genomic ordered by chromosomes and positions. SNPs reaching genome-wide significance, defined as p≤ 5×10^−8^, are visualized above the red line.

### GWAS identifies ROP-associated SNPs with genome-wide significance or suggestive significance correlating with both novel and established ROP risk genes

As noted in Table 2, we identified 10 loci which demonstrate genome-wide significance (p≤ 5×10^−8^) or suggestive significance (p≤ 5×10^−6^) of association with ROP severity, defined by stage 3 disease or greater, both with and without correction for birth weight (BW) and gestational age (GA). The position, effect allele and frequency, odds ratio, p value and nearest gene is delineated in Table 2. SNPs demonstrating the lowest p value in each locus/region were selected and the bioinformatically determined nearest gene was used. Under the additive genetic model, the ROP risk corresponds to the number of copies of the effect allele when the odds ratio is greater than 1. As noted, SNPs rs2058019 (*GLI3* gene) and rs11563856 (*CLDN12* gene) both reach genome wide significance, although only rs2058019 remains significant at this level when controlling for the potentially confounding effects of BW and GA. In two of the loci meeting suggestive significance, the nearest gene has a prior association with ROP disease in either humans or animal models as noted by bolded text. Finally, we performed an *in silico* analysis to determine if each listed SNP had a described expression quantitative trait loci (eQTL) association in the EyeGEx^27^ database. We identified a number of established regulatory associations for our top SNPs as detailed in Table 2 within the retina, further supporting relevance to the ocular microenvironment.

### rs2058019 contributes to genetic susceptibility for ROP in diverse populations

Given established differences in clinical ROP risk based on race and ethnicity^22,23^, we analyzed the association of the lead *GLI3* SNP, rs2058019, stratified by these variables. As seen in Table 3, the association between rs2058019 and ROP disease is most significant when considering all patients, although odds ratios and allele frequencies between cases and controls demonstrate that the degree and direction of association remain consistent for Caucasian and Hispanic subjects. Interestingly, the African American subjects do not appear to be contributing to this association. This latter finding does not reach significance in this sample and thus requires validation within a larger population. As noted in Supplemental Table 3, we did not find suggestive evidence for significant differences is MAF or direction of effect within racial or ethnic populations for other top SNPs.

### Multiple significant SNPs are identified regionally related to *GLI3*

**Figure 2:**
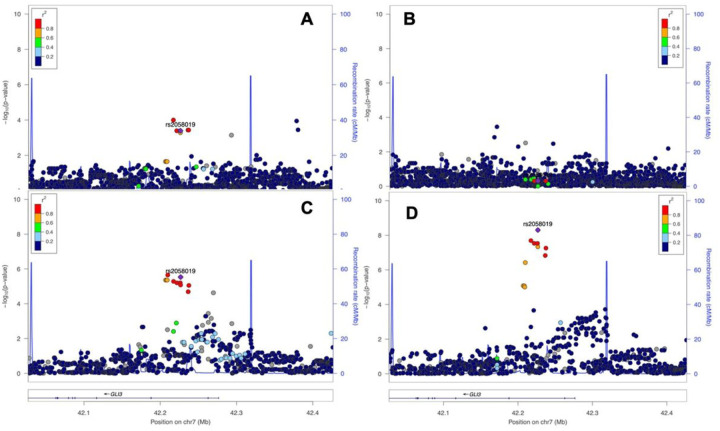
Regional association plots of top genome-wide significant SNP rs2058019 (*GLI3* gene) among each ethnic group: A: Caucasian (CA); B: African American (AA), C: Hispanic (HA), and D: all combined samples (ALL).

To determine the regional significance for the *GLI3* pan-locus, we plotted all SNP-associations within 100 Kb of the *GLI3* gene for all patients as well as for racial and ethnic subsets. As pictured in Figure 2, a regional plot with the Y axis depicting the −log transformed p value and the X axis showing chromosomal position, we illustrate the *GLI3* gene regional association for all ethnic and racial groups with ROP severity. The lead SNP rs2058019 was identified as a top-associated SNP within the pan-locus for the Caucasian and Hispanic groups and both the best and sentinel SNP when all patients were considered together. Significant regional SNPs for each group are delineated fully in Supplemental Table 4. Also pictured, we determined the linkage disequilibrium (LD) for each SNP, defined by the r^2^ using the included color scheme. LD was determined between SNPs and genes involved/close to SNPs identified.

### GWAS identifies ROP-associated SNPs with genome-wide significance or suggestive significance correlating with both novel and established ROP risk genes

As noted in Table 2, we identified 10 loci which demonstrate genome-wide significance (p≤ 5×10^−8^) or suggestive significance (p≤ 5×10^−6^) of association with ROP severity, defined by stage 3 disease or greater, both with and without correction for birth weight (BW) and gestational age (GA). The position, effect allele and frequency, odds ratio, p value and nearest gene is delineated in Table 2. SNPs demonstrating the lowest p value in each locus/region were selected and the bioinformatically determined nearest gene was used. Under the additive genetic model, the ROP risk corresponds to the number of copies of the effect allele when the odds ratio is greater than 1. As noted, SNPs rs2058019 (*GLI3* gene) and rs11563856 (*CLDN12* gene) both reach genome wide significance, although only rs2058019 remains significant at this level when controlling for the potentially confounding effects of BW and GA. In two of the loci meeting suggestive significance, the nearest gene has a prior association with ROP disease in either humans or animal models as noted by bolded text. Finally, we performed an *in silico* analysis to determine if each listed SNP had a described expression quantitative trait loci (eQTL) association in the EyeGEx^27^ database. We identified a number of established regulatory associations for our top SNPs as detailed in Table 2 within the retina, further supporting relevance to the ocular microenvironment.

### rs2058019 contributes to genetic susceptibility for ROP in diverse populations

Given established differences in clinical ROP risk based on race and ethnicity^22,23^, we analyzed the association of the lead *GLI3* SNP, rs2058019, stratified by these variables. As seen in Table 3, the association between rs2058019 and ROP disease is most significant when considering all patients, although odds ratios and allele frequencies between cases and controls demonstrate that the degree and direction of association remain consistent for Caucasian and Hispanic subjects. Interestingly, the African American subjects do not appear to be contributing to this association. This latter finding does not reach significance in this sample and thus requires validation within a larger population. As noted in Supplemental Table 3, we did not find suggestive evidence for significant differences is MAF or direction of effect within racial or ethnic populations for other top SNPs.

### Multiple significant SNPs are identified regionally related to *GLI3*

To determine the regional significance for the *GLI3* pan-locus, we plotted all SNP-associations within 100 Kb of the GLI3 gene for all patients as well as for racial and ethnic subsets. As pictured in Figure 2, a regional plot with the Y axis depicting the −log transformed p value and the X axis showing chromosomal position, we illustrate the *GLI3* gene regional association for all ethnic and racial groups with ROP severity. The lead SNP rs2058019 was identified as a top-associated SNP within the pan-locus for the Caucasian and Hispanic groups and both the best and sentinel SNP when all patients were considered together. Significant regional SNPs for each group are delineated fully in Supplemental Table 4. Also pictured, we determined the linkage disequilibrium (LD) for each SNP, defined by the r^2^ using the included color scheme. LD was determined between SNPs and genes involved/close to SNPs identified.

### Top-identified SNP associations vary by race and ethnicity

To further determine race and ethnicity-based differences in SNP associations with ROP severity, we performed our GWAS analysis separately for each racial and ethnic group. As pictured in Supplemental Figure 2, which visually represents SNP associations for each population, racial and ethnic differences are present. As seen in Table 4, we find several SNP associations which are racially or ethnically specific and reach genome-wide or suggestive level significance. Notably, rs9978278 (*CLDN14* gene) and rs74048122 reach genome-wide significance, which remains significant after correction for BW and GA. Although the number of observations is decreased when analyzing by race and ethnicity, these findings suggest possible differences in genetic risk for ROP by population ancestry.

### SNPs with suggestive or greater association with ROP severity in the full iROP cohort demonstrate cross-significance within the Hispanic Diabetic Retinopathy GOLDR cohort

Our study design, which identifies SNPs associated with ROP disease stage 3 or greater, enriches our findings for genetic variation associated with pre-retinal neovascular pathobiology. Diabetic retinopathy (DR) is another form of retinal disease characterized by pre-retinal neovascularization. Therefore, to validate the significance of *GLI3* genetic variation to pre-retinal neovascular disease, we performed an extension analysis of top-associated iROP SNPs, those with p ≤5×10^−6^ or greater significance levels, and SNPs regionally related for DR association within the Genetics of Latino Diabetic Retinopathy (GOLDR) GWAS dataset. Importantly, the GOLDR population consists of Hispanic patients with diabetic retinopathy (DR)^28,29^. As noted in Table 5, we found cross-significance for a number of SNPs with DR in GOLDR and with ROP severity in our iROP GWAS cohort. This included SNPs within *GLI3, DCLK1, SP4, PTPRD,RPL30* and *RIDA*.

### ROP Genetic Risk Score (GRS) demonstrates increased significance over single SNP associations

Joint estimation of SNP effects has been suggested as a superior approach to improve SNP-based disease prediction and risk stratification, including in eye disease by our group^30–35^. We calculated GRSs (Score A, Score B) incorporating SNPs based on the SNP selection by LASSO method to determine if combined SNP profiles were more highly associated with ROP severity^36^. ROP severity was represented as an ordinal categorial variable denoting stage including none, stage 1, stage 2, etc for this analysis. Table 6 compares the association results between different GRSs. Score A consists of the top *GLI3* SNPs as well as 45 other top ROP-associated SNPs and Score B consists of 255 top-ROP associated SNPs. As depicted in Table 6, each GRS was significantly associated with ROP severity. To determine the proportion of variation within the phenotype which can be explained using each risk score, and therefore the breadth of ROP phenotype that is associated with each GRS, we analyzed adjusted R-squared. As noted, score A accounted for a larger adjusted R-squared than the top SNP alone (0.63 versus 0.34), suggesting that the additional SNPs in this risk score together account for a more variance in ROP phenotype/severity than the *GLI3* SNP alone. The drop in adjusted R-squared between Score A (0.63) and Score B (0.45) is likely due to the addition of noise in the model resulting from adding SNPs in this score and over-fitting. Taken together, the GRS demonstrate stronger associations with all ROP phenotypes.

### Significant retinal vascular SNPs demonstrate relevance to ROP risk

To determine the relevance of our dataset to vascular pathology more broadly, we analyzed the association of significant retinal vascular SNPs, identified in the central retinal venule equivalent (CRVE) and the central retinal arteriole equivalent (CRAE) dataset^37^, within our iROP dataset. Using the summary statistics from the exome chip meta-analysis for CRVE and CRAE, we tested all CRVE/CRAE loci with p ≤ 0.01 for ROP association within our iROP dataset. A total of 583 SNPs from the CRVE/CRAE dataset were available for analysis in our iROP dataset. After Bonferroni correction for multiple testing, we identified significant associations for SNPS rs13079478 (*FYCO1* gene), rs33910087 (*FYCO1* gene) and rs12357206 (*ANK3* gene) with ROP severity as noted in Table 7.

### Extension of associations for severe ROP SNPs identified in a candidate gene study replicate significance of the *RELN* gene

To independently replicate previously identified ROP-associated SNPs, we evaluated candidate SNPs found to be significantly associated with severe ROP by Harnett et al.^17^ within our iROP GWAS dataset. As noted in Table 8, rs10251365 in the gene *RELN* is significantly associated with ROP severity in both cohorts (p=0.009). The p values of SNP-associations with ROP severity based on stage using our data are included for comparisons and confirmation.

### GLI3 and other top associated genes are expressed in human retinal and retinal pigment epithelial tissues (RPE).

To determine the relevance of *GLI3* genetic variation to retinal pathophysiology, we sought to determine the expression patterns of GLI3 within human retinal and RPE tissues. Human donor macular or peripheral retinal and RPE tissues from control eyes (n=10), average age 72 years, were analyzed using RNA-sequencing. Expression was analyzed using DESeq2^38^ as we have previously published^39^. Analyses were done for genes relative to our top-associated ROP SNPs as listed in Table 2. All top genes except PRPF4B were expressed in human donor macular tissues; the tissue type with greatest expression and fold difference between tissue types (neurosensory retina or RPE) is listed in Table 9. As noted, GLI3 was expressed in both the neurosensory retina and RPE; macular RPE expression was significantly greater by 2.54-fold (p=3.3 × 10 −15, after correction for multiple testing using Benjamini-Hockberg) than neurosensory retinal expression (Table 9). We also analyzed differential GLI3 expression between macular and peripheral RPE tissues to determine the region with greatest expression. Interestingly, we found that GLI3 expression was 1.72 fold higher in the peripheral RPE as compared to the macular RPE (p<0.01) which is notable given the presence of ROP most commonly within the peripheral retina.

## Discussion

Herein, we report the first successful GWAS for ROP which identifies a novel ROP risk association for the *GLI3* SNP rs2058019, reaching genome-wide significance within a multiethnic population. This is the first reported genome-wide significant SNP association for ROP, which shows an association trend in the same direction for both Hispanic and Caucasian infants and therefore, may demonstrate disease applicability across populations. We further identify significance of GLI3 variation for preretinal neovascular disease and established ROP genetic risk associations using extension analysis within an independent replication cohort consisting of Hispanic individuals with diabetic retinopathy and severe ROP candidate dataset^17^ respectively. Finally, we demonstrate relevance of our findings to human retinal and RPE tissues using expression profiling within donor eyes. Owing to the multiethnic composition of our cohort, our analysis also is the first to demonstrate racial and ethnic differences in GWAS-determined SNP associations. While our lead SNP, rs2058019, demonstrates a less significant association within the African American populations, as noted in Table 4, we identify several SNPs with significant association within only the African American population compared with the Hispanic or non-Hispanic Caucasian populations. This is consistent with, and indeed may speak to, observed clinical differences in ROP incidence and severity, including greater disease development and severity in Caucasian populations^22,23^. However, while suggestive of disparate genetic risk for ROP disease within different racial and ethnic populations, these results require replication in a larger population to clarify ethnic and racial differences.

GLI3 has not previously been associated with ROP disease, though it has a defined role within ocular development. Specifically, the dual role for Gli3 as both a transcriptional activator and repressor of canonical sonic hedgehog (Shh) signaling, has been found to control differentiation of the RPE and rod photoreceptor layer during Xenopus as well as Medaka fish eye development^40,41^. Further, abnormal expression of the Hedgehog signaling pathway has been shown in ROP within the oxygen induced retinopathy (OIR) murine model^42^. More broadly Gli3 has been shown to regulate both the innate and adaptive immune response, with described roles in fetal CD4 and CD8 thymocyte and T-cell development^43,44^. Certainly, pre-retinal neovascular disease, including within ROP and diabetic retinopathy, has been associated with aberrant inflammation^45–47^. Thus, there is a feasible relationship between GLI3 and ROP disease incidence and severity.

The lead SNP, rs2058019, is present within an intronic region and has not previously been associated with disease. Thus, the functional significance is not clear, although aberrancy in *GLI3* is associated with human disease, including polydactly syndromes^48^ and malignancy^49–52^. Functionally, both aberrant GLI3 repressor and activator roles have been associated with underlying patho-mechanisms, resulting in imbalance within epithelial to mesenchymal transition^50,52,53^, AKT/ERK1 activation^54^, p53 function^55^ and autophagy^56^. Significant differences in GLI3 expression between control and diseased tissues is also thought to underlie pathobiology as described in AML^57^ and colon cancer^55^, in some cases regulated by differential methylation^53,57^. Thus, there is significant precedent for aberrancy of *GLI3* DNA structure, expression, or function in human disease. Future work will seek to determine the functional significance of this SNP association to ROP disease development in preterm infants. Our work demonstrating GLI3 expression within adult human donor eyes within both neurosensory retina and RPE/choroid tissues with a higher gradient within the RPE/choroid, supports the premise that GLI3 has relevance to human pre-retinal neovascular disease.

In addition to *GLI3*, genes associated with top identified SNPs within our dataset, as demonstrated in Table 2, have shown associations with ROP in other work. ARHGEF7, demonstrates significant differential expression within rodent models of pre-retinal neovascularization and is also different between early and late stages of the disease within the OIR model of ROP^58^. There is also precedent for DPP4 function within retinal vascular homeostasis which is perturbed in murine models of both ROP and diabetic retinopathy. Within the OIR model, DPP4-inhibition increased retinal vascularity and leakage while in the murine diabetic retinopathy model, DPP4-inhibition increased retinal vascular leakage^59^. These murine findings are substantiated in human literature, which demonstrates increased progression of proliferative diabetic retinopathy in patients taking DPP4-inhibitors^60^. The inverse was also found to be true, namely that topical administration of dipeptidyl peptidase prevents vascular leakage in a diabetic mouse model^61^. Finally, while CLDN12 has not been found to play a role in pre-retinal neovascular disease or ROP specifically, the Claudin family of proteins have precedent in neovascular ocular disease^62,63^. This is particularly interesting given the identification of another Claudin family member, CLDN14, with a significant SNP association for ROP severity in African Americans (Table 4).

Genes associated with our top-identified SNPs also support findings from case control candidate-approach SNP-chip studies. As noted in Table 8, extension of associations for the top 10 SNPs identified for severe ROP by Hartnett *et al*.^17^ demonstrates independent replication for rs10251365 in the *RELN* gene (p=0.009). While additional cross-significance was not identified, the study design, GWAS versus candidate approach, and outcome measures, “severe compared to mild ROP” versus ROP severity by stage, differ between these studies. As noted above, we also identified the greatest association for these top SNPs within a multiethnic population which is distinct from the roughly 70% Black race population examined by Hartnett et al. Thus, the lack of additional overlap may also be informed by racial and ethnic differences between studied populations or may reflect other aspects of ROP risk, such as timing of ROP presentation as well as susceptibility of disease progression given external stresses that interact with race and ethnicity. As noted, we identify unique genetic ROP risk for African American versus Caucasian or Hispanic populations. While African American unique SNPs were identified in a smaller proportion of our cohort, and therefore based in fewer observations, these findings warrant further examination within larger populations. Future meta-analysis may allow for better harmonization and additional independent validation. Taken together, our identified SNP associations demonstrate relevance to described ROP patho-mechanisms, while also identifying novel associations for further study.

The overall relevance of genetic variation to ROP risk has been debated^64^. Our work substantiates genetic ROP risk which is further highlighted by our genetic risk score assessment. This analysis methodology has been used in numerous disease contexts, including by our group^34,65^, to assess the magnitude of genetic risk for disease outcome measures. In our analysis, ROP disease severity was more significantly associated with polygenic SNP subsets than with the most significant individual GWAS SNPs. This suggests the existence of more extensive genetic variation contributing to ROP and thus, the overall importance of this continued approach.

Finally, the relevance of our data to vascular pathobiology more broadly is evidenced in our extension analyses within independent replication datasets from other forms of pre-retinal neovascular (DR/GOLDR) and retinal vascular (CVRE/CRAE) pathobiology. We identify SNP associations that “bridge” these disease contexts and thus may speak to co-occurring mechanisms of disease. Among these, SNP-associations at the *GLI3, DCLK1, SP4, PTPRD,RPL30* and *RIDA* genes were replicated in an independent Hispanic diabetic retinopathy (DR) cohort and thus shared within the context of pre-retinal neovascular disease which is present in both ROP ≥ stage 3 and proliferative diabetic retinopathy. While DCLK1^66^ and PTPRD^67^ have been previously associated with DR in animal and human studies respectively, the remaining associations are novel within the context of pre-retinal neovascular disease and represent areas of future study. Similarly, our identification of SNPs within *FYCO1* and *ANK3* as shared between vascular pathobiology in the CVRE/CRAE dataset and our iROP cohort, represents novel associations. Both genes have associations within the eye and retina, though most significantly with anterior segment pathology including infantile cataract and coloboma^68,69^.

Taken together, we report the largest ROP GWAS to date, which identifies a novel locus at *GLI3* on chromosome 7 demonstrating genome wide level significance, the first ROP associated variant to do so. We further identify 7 additional loci with suggestive level significance for ROP severity. We demonstrate relevance for GLI3 and other top-associated genes to findings from prior studies and human ocular disease through *in-silico* extension analyses, genetic risk score analysis and expression profiling in human donor eye tissues. Significance varied by race, with greater association measured within Caucasian and Hispanic compared with African American infants. This may underlie clinically observed differences in ROP development which are also represented in our cohort which demonstrates African American ROP prevalence of 43% compared to 52% and 67% for Caucasian and Hispanic populations respectively. Replication of this study using a larger and more diverse population would allow for validation of these findings, particularly with regard to differences in genetic ROP risk relative to ethnicity and race^70–72^. In summary, we report the largest ROP GWAS to date, identifying a novel locus at *GLI3* with relevance to retinal biology supporting genetic susceptibilities for ROP risk with possible variability by race and ethnicity.

## Methods

Recruited Study Cohort: The Imaging and Informatics for ROP (iROP) study is a multicenter, prospective, ROP cohort study collecting clinical, biologic, and imaging data from preterm infants born with GA and BW risk for ROP as previously published by our group^16,25^. For all enrolled infants, retinal images were obtained using a wide-angle fundus camera [RetCam; Natus Medical Incorporated, Pleasanton, CA]; biologic specimens included blood or saliva and was obtained with specific consent. Informed consent was provided to the parent/guardian under approved IRB protocols at each study site which conformed to the tenets of the Declaration of Helsinki. When consent was given for biologic sample collection blood was collected in a purple top microtainer tube. White blood cells within the buffy coat were isolated using a standard protocol and DNA extracted using a Qiagen Allprep system for subsequent genetic analysis. All images were deidentified in accordance with HIPPA privacy rules. ROP phenotype was determined for each eye by consensus from at least 3 trained graders with ROP expertise employing image-based diagnoses and the clinical exam diagnosis at each study center as previously described^26^. ROP severity was graded based on the worst stage present for either eye. The iROP database was reviewed to identify infants with both biologic and phenotypic data availability which included 920 infants. Patients without detailed demographic, ROP screening, clinical, or imaging data were excluded.

### Genotype, quality control and imputation

Genotyping was performed using genotyping arrays from Illumina Infinium Global Screening Array (GSA, Santa Clara, CA, USA). Principal component analysis (PCA) was performed using SMARTPCA implemented in EIGENSOFT^73^. Standard quality control procedure was performed for all genotype samples/SNPs. For example, samples with genotyping rate less than 0.92, gender mismatches, PCA outliers defined as greater than 3 standard deviations of top principal component variables (PCs), SNPs with genotyping rate less than 0.95 and Hardy-Weinberg Equilibrium (HWE) p value less than 10–6, were excluded for analysis. Imputation was conducted by Minimac using the Michigan Imputation Server^74^ with the reference panel from the 1000 Genomes Project Phase 3 Haplotypes^75^. Autosomal SNPs with the minor allele frequency (MAF) greater than 0.05 and imputation quality (rsq) greater than 0.3 were analysed.

### GOLDR cohort (Genetics of Latino Diabetic Retinopathy, GOLDR)

The GOLDR study is a family-based study assessing diabetes and diabetic complications in families (siblings and/or parents) of a proband, defined as having type 2 diabetes and either known DR or a diabetes duration of ≥10 years^28^. Participants are all Latinos of Mexican or Central American origin, recruited, and studied between 2007 and 2012 at the Lundquist Institute (formerly called the Los Angeles BioMedical Research Institute) at Harbor-UCLA Medical Center (HUMC). In total, there were 612 participants with type 2 diabetes from 216 families, with sizes ranging from 1 to 8 members per family in the study. These samples were genotyped with the Illumina Cardio-Metabochip and imputed with 1000 Genome using Michigan Imputation Server.

### Statistical Methods

#### Genome-wide association tests for single SNPs

Under the additive genetic model, we performed GWAS tests with allele dosage for all genotyped and imputed SNPs stratified by each ethnic group, and also combined, using Efficient Mixed Model Association eXpedited (EMMAX) method^76^ implemented in EPACTS (https://genome.sph.umich.edu/wiki/EPACTS). Outcome measure for association was ROP severity defined by stage 3 or higher. Birth weight, gestational age, gender, top 3 PCs, and the relatedness by the genomic relationship matrix estimated using the genomic data, were included as covariates. Associations with p < 5×10^−8^ were considered GWAS significant. To further investigate the relationship between birth weight/gestational age and ROP association signals, we also performed the association tests with and without adjustment for birth weight/gestational age. In addition, GWAS analyses stratified by each ethnic or racial group were also performed in the same way.

#### Retinal expression quantitative trait loci (eQTL) Analysis:

To investigate the relationship between association signals and gene expression, we evaluated whether top ROP SNPs identified from GWAS are related to eQTL in the genotype-tissue specific expression databases, EyeGEx^27^, by far the largest reference tissue bank for gene expression and regulation in the human retina.

#### Genetic risk score (GRS) of ROP:

Three genetic risk scores were tested. For the “GLI3” risk score was composed of the genotype for the most significant SNP, rs2058019. In order to reduce overfitting of the genetic risk score to ROP stage due to the large number of SNPs relative to the number of subjects, we fit a linear regression model with a LASSO penalty using the R package “glmnet” with 451,565 directly genotyped SNPs^77,78^. The outcome variable was ROP Stage as an ordinal categorial variable (None, Stage 1, Stage 2 and Stage 3 and above). The glmnet software performs 10-fold cross-validation of the mean-squared error with a series of 100 penalties selected by a coordinate-descent; two models result, SNP score B was composed of 255 SNPs selected at the minimum penalty, and SNP score A was composed of 49 SNPs at 1 standard error (1 s.e.) from the mean-squared error. Selecting SNPs at 1 s.e. is an additional way to reduce overfitting. SNP score B and SNP score A were the sum of the number of risk alleles across all the SNPs for that score. Scores were tested in a linear model with sex, birthweight, gestation age, and the first two principal components of the SNP data. Principal components were calculated using a singular value decomposition.

#### Extension of the association signals for diabetic retinopathy in GOLDR:

Since many infants are Hispanic origin, we further performed the SNP association tests in an independent Hispanic cohort (GOLDR). Using diabetic retinopathy (DR) patients as cases (72 cases vs 491 controls), the association tests were performed for the top significant GWAS and suggestive ROP association genes/loci identified above (10 loci) in GOLDR cohort with the logistic regression. The significance threshold for the extension analysis was defined by Bonferroni correction with the number of genes/loci tested (0.05/10=0.005).

#### Extension of association signals for CRVE/CRAE loci:

Further extension analysis was performed to cross-check the overlapping SNP-associations with CRVE and/or CRAE. Using the summary statistics from the exome chip meta-analysis for CRVE and CRAE^37^, we evaluated association signals for all available CRVE/CRAE loci with p values<0.01 (583 SNPs in total) in both ROP dataset. The significance threshold for the extension analysis was defined by Bonferroni correction with the number of SNPs tested (0.05/583=9×10^−5^).

#### Extension of the association signals for severe ROP identified by Hartnett et al. candidate gene study:

In order to replicate the ROP association signals by other studies, we tested the significant SNPs identified by Harnett et al^17^ associated with severe ROP. Out of 22 top severe ROP signals, we evaluated 15 SNP-associations available in our analysis. The significance threshold for the extension analysis was defined by Bonferroni correction with the number of SNPs tested (p=0.05/15=0.0033).

Donor Eye Tissue Repository: Methods for human donor eye collection were previously described in detail according to a standardized protocol^79^. In brief, in collaboration with the Utah Lions Eye Bank, donor eyes were procured within a 6-hour post-mortem interval, defined as death-to-preservation time. Both eyes of the donor underwent post-mortem phenotyping with ocular imaging, including spectral domain optical coherence tomography (SD-OCT), and color fundus photography as published. Retinal pigment epithelium/choroid was immediately dissected from the overlying retina, and macula separated from periphery using an 8mm macular punch. For both peripheral and macular tissues, RPE/choroid was separated from the overlying retinal tissue using microdissection; tissue planes were optimized to minimize retinal contamination of RPE/choroid samples using a subsequent 6mm RPE/choroid tissue punch. Isolated macular and peripheral RPE/choroid samples were preserved in RNAlater (Ambion, ThermoFisher, Waltham, MA, USA), and stored at −20°C for 24 hours then transferred to −80°C. Phenotype analysis was performed as described^79^ by a team of 4 retinal specialists and ophthalmologists at the University of Utah School of Medicine, Moran Eye Center and the Massachusetts Eye and Ear Infirmary Retina Service. Agreement of all 4 specialists upon independent review of the color fundus and OCT imaging was deemed diagnostic; discrepancies were resolved by collaboration between a minimum of three specialists to ensure a robust and rigorous phenotypic analysis. One eye was per donor was biochemically analyzed. Institutional approval, and the consent of patients to donate their eyes and for research purposes was obtained from the University of Utah and conformed to the tenets of the Declaration of Helsinki. All tissue was deidentified in accordance with HIPPA privacy rules.

### Nucleic acid extraction and RNA-Sequencing:

Transcriptional profiling of macular and peripheral retina and RPE/choroid tissues from 10 unrelated control eye tissue donors, average age 72 years, was performed using RNA-sequencing. DNA and RNA were extracted from peripheral and macular neurosensory retinal and RPE/choroid tissues, prepared as described above, using the Qiagen All-prep DNA/RNA mini kit (cat #80204) per the manufacturer’s protocol. Quality of RNA samples was assessed with an RNA Nano Chip (Agilent). Total RNA was poly-A selected and cDNA libraries were constructed using the Illumina TruSeq Stranded mRNA Sample Preparation Kit (cat# RS-122-2101, RS-122-2102) according to the manufacturer’s protocol. Sequencing libraries (18 pM) were chemically denatured and applied to an Illumina TruSeq v3 single read flow cell using an Illumina cBot. Hybridized molecules were clonally amplified and annealed to sequencing primers with reagents from an Illumina TruSeq SR Cluster Kit v3-cBot-HS (GD-401-3001). Following transfer of the flowcell to an Illumina HiSeq instrument (HCS v2.0.12 and RTA v1.17.21.3), a 50-cycle single read sequence run was performed using TruSeq SBS v3 sequencing reagents (FC-401-3002)

### Primary Analysis of RNA Sequencing Data:

Each of the 50bp, poly-A selected, non-stranded, Illumina HiSeq fastq datasets were processed as follows: Reads were aligned using NovoCraft’s novoalign 2.08.03 software (http://www.novocraft.com/) with default settings plus the -o SAM -r All 50 options to output multiple repeat matches. The genome index contained human hg19 chromosomes, phiX (an internal control), and all known and theoretical splice junctions based on Ensembl transcript annotations. Additional details for this aspect of the protocol are described elsewhere (http://useq.sourceforge.net/usageRNASeq.html).Next, raw novoalignments were processed using the open source USeq SamTranscriptiomeParser (http://useq.sourceforge.net) to remove alignments with an alignment score greater than 90 (~ 3 mismatches), convert splice junction coordinates to genomic, and randomly select one alignment to represent reads that map equally well to multiple locations. Relative read coverage tracks were generated using the USeq Sam2USeq utility (http://useq.sourceforge.net/cmdLnMenus.html#Sam2USeq) for each sample and sample type (Normal Retina, Neovascular AMD Retina, Intermediate AMD Retina, Normal RPE, Neovascular AMD RPE, and Intermediate AMD RPE). These data tracks are directly comparable in genome browsers and good tools to visualize differential expression and splicing. Estimates of sample quality were determined by running the Picard CollectRnaSeqMetrics application (http://broadinstitute.github.io/picard/) on each sample. These QC metrics were then merged into one spreadsheet to identify potential outliers. Agilent Bioanalyzer RIN and library input concentration columns were similarly added for QC purposes (http://www.genomics.agilent.com).

## Figures and Tables

**Table 1:** iROP Infants Included in GWAS Analysis

Race/Ethnicity	No ROP	Stage 1	Stage 2	Stage 3	Stage 4	Stage 5	Total
Caucasian	129	57	72	58	10	0	326
African American	60	17	20	13	0	1	111
Hispanic	128	52	127	65	23	15	410
Other	34	11	16	11	0	1	73
Total %	351 (38.2%)	137 (14.9%)	235 (25.5%)	147 (16%)	33 (3.6%)	17 (1.8%)	920

**Table 2:** Association variants identified for all ROP samples with p <5×10^−6^.

SNP	Chr	Pos	Effect Allele	AF	rsq	OR	p-value	p-value adj for BW & GA	gene	eQTL p-value
rs2058019	7	42226712	T	0.06	0.96	1.27	5.25E-08	4.96E-09*	GLI3	NA
rsl1563856	7	90141855	G	0.17	0.91	1.16	5.84E-08	1.71E-07	CLDN12	>0.05
rs62052253	16	90048889	T	0.22	0.81	1.13	9.67E-07	4.50E-07	DEF8. TUBB3	2.7E-04
rs61948265	13	36559030	A	0.14	0.68	1.14	7.90E-06	6.50E-07	DCLK1	0.02
rs141411503	7	21465569	T	0.06	0.89	1.21	3.51E-05	8.38E-07	SP4	0.01
rs78971944	13	111811973	G	0.09	0.87	1.17	8.70E-06	1.83E-06	**ARHGEF7**	>0.05
rs9644892	9	8800078	G	0.36	0.93	1.10	1.40E-06	2.14E-06	PTPRD	7.7E-03
rs72870405	2	162937083	A	0.12	0.96	1.14	1.13E-06	3.56E-06	**DPP4**	>0.05
rs2306129	8	99105726	C	0.39	0.84	1.09	6.04E-05	3.77E-06	ERICH5. RPL30. RIDA	1.90E-21
rs7751076	6	3980472	A	0.08	0.93	1.17	9.37E-04	5.52E-06	PRPF4B	>0.05

**Table 3:** Lead SNP rs2058019 demonstrates association with severe ROP disease in Hispanic and Caucasian populations but not in African American populations

Ethnicity/Race	Effect Allele	AF: Case	AF: Control	AF: All	OR	P
HA	T	0.22	0.08	0.11	1.26	2.95E-06
CA	T	0.04	0.01	0.01	1.61	4.0E-04
AA	T	0	0.02	0.01	0.87	0.49
All	T	0.14	0.04	0.06	1.27	4.96E-9

HA: Hispanic Americans; CA: Caucasian Americans; AA: African Americans; All: all subjects combined.

AF: allele frequency of effect allele.

**Table 4:** Racial and Ethnic differences in GWAS-identified SNP associations with severe ROP disease

SNP	chr:position	Ethnicity	Effect allele	AF: Case	AF: Control	OR	p-value	p-value adj. BW &GA P	gene
rs 17048572	4:137201813	HA	C	0.02	0.02	Na	Na	na	none
		CA	C	0.14	0.04	1.39	4.19E-5	1.99E-8*	
		AA	C	0.04	0.06	0.88	0.40	0.15	
		All	C	0.06	0.04	Na	Na	na	
rs 9978278	21:37894721	HA	T	0.06	0.09	0.96	0.88	0.48	CLDN14
		CA	T	0.12	0.24	0.94	5.8E-03	0.08	
		AA	T	0.25	0.03	1.71	4.04E-9*	1.63E-8*	
		All	T	0.09	0.13	0.97	0.16	0.24	
rs74048122	14:43889545	HA	G	0.03	0.06	Na	Na	na	None
		CA	G	0.08	0.07	0.99	0.55	0.86	
		AA	G	0.32	0.04	1.53	2.62E-8*	3.14E-8*	
		All	G	0.07	0.06	1.03	0.25	0.43	
rs 1004464	9:131003677	HA	G	0.06	0.13	0.93	0.17	0.14	DNM1
		CA	G	0.13	0.18	0.95	0.40	0.20	
		AA	G	0.61	0.19	1.29	1.74E-7	2.62E-8*	
		All	G	0.14	0.17	0.99	0.86	0.82	

**Table 5.** Cross-significance of top SNPs for Retinal Pathology Characterized by Pre-retinal Neovascular Disease including ROP and Diabetic Retinopathy (GOLDR)

Gene	SNP	Chr	position	GOLDR		ROP	
				odds ratio	P value	odds ratio	P value
GLI3	rs74527981	7	42205730	2.22	9.3E-04	na	Na
	rs17172024	7	42210112	2.22	9.3E-04	na	Na
	rs2058019	7	42187113	ns	Ns	1.27	4.90E-09
CLDN12	No loci associated with DR for whole region						
DEF8, TUBB3	No loci associated with DR for whole region						
DCLK1	rs35596426	13	36452952	5.18	4.3E-04	Na	Na
	rs9576059	13	36452266	4.83	5.9E-04	Na	Na
	rs61948265	13	35984893	ns	Ns	1.14	6.50E-07
SP4	rs11770747	7	21552122	1.96	7.6E-04	ns	Ns
	rs78539005	7	21547126	2.51	8.5E-04	1.09	0.03
	rs141411503	7	21425951	ns	Ns	1.21	8.38E-07
ARHGEF7	No loci associated with DR for whole region						
PTPRD	rs79715438	9	8809579	3.48	5.0E-04	na	Na
	rs75240118	9	8809921	3.48	5.0E-04	na	Na
	rs80041124	9	8810631	3.48	5.0E-04	na	Na
	rs76777639	9	8488087	3.30	9.6E-04	na	Na
	rs529032346	9	8499029	3.30	9.6E-04	na	Na
	rs9644892	9	8800078	ns	Ns	1.1	2.14E-06
DPP4	No loci associated with DR for whole region						
RPL30, RIDA	rs2514337	8	98113613	0.49	5.4E-04	0.94	4.4E-04
	rs2447504	8	98113678	0.49	5.4E-04	0.94	4.4E-04
	rs2306129	8	98093498	0.66	0.04	1.09	3.77E-06
PRPF4B	No loci associated with DR for whole region						

ns: not significant.

na: association tests not applicable for SNPs with MAF less than 0.05.

**Table 6.** Association tests using genetic risk score (GRS) method.

GRS	ROP(iROP).		
	Beta	r2	P
SNP Score A (49 SNPs)	0.14	0.63	<2e-16
SNP Score B (255 SNPs)	0.05	0.45	3.8e-16
GLI3 only	0.37	0.34	3.8e-7

**Table 7.** Extension of CRVE/CRAE significant loci to iROP dataset

SNP	Gene	iROP	CRVE/CRAE
		P	P
rs13079478	FYCO1	7.96e-6	3.0E-03
rs33910087	FYCO1	1.93e-5	3.0E-03
rs12357206	ANK3	5.07e-5	9.0E-05

summarizes SNPs (identified for CRVE/CRAE from the literature) confirmed to be significantly associated with ROP Stage.

**Table 8.** Association of SNPs identified with severe ROP.

SNP	MaF	gene	P for severe ROP (Hamett, 2014)	P for ROP ≥ Stage 3 (iROP)
rs9332681	Na	*F5*	0.99	Na
rs379489	0.287	*CFH*	3.8E-03	0.I4
rs395544	0.287	*CFH*	5.4E-03	0.14
rs11587174	0.002	*FI3B*	0.87	na
rs1467199	0.211	*STATI*	0.82	0.81
rs34417936	0.014	*IL*	0.66	Na
rs2299386	0.380	*RELN*	1.6E-03	0.73
rs10251365	0.332	*RELN*	1.0E-03	0.0092*
rs168879811	0.309	*NRGI*	4.0E-04	0.28
rs168879814	0.311	*NRGI*	3.0E-04	0.25
rs2353512	0.002	*BDNF-AS*	0.77	Na
rs7127507	0.295	*BDNF*	2.0E-04	0.70
rs2049046	0.479	*BDNF*	3.00E-05	0.41
rs7934165	0.471	*BDNF*	2.00E-05	0.53
rs12281784	0.101	*LRP4*	0.54	0.14
rs9989002	0.211	*IGFI*	2.2E-03	0.81
rs11620315	0.149	*FLTI*	0.45	0.76
rs1319859	0.424	*IGFIR*	0.12	0.04
rs884636	0.040	*PCPEPIL*	0.10	na
rs7204874	Na	*NSMC EIjIL4 R*	1.4E-03	na
rs2057768	0.287	*IL4R*	7.0E-04	0.65
rs1551005	Na	*TTR*	0.96	na

MAF: minor allele frequency.

na: SNP not available in ROP dataset and/or with MAF less than 0.05.

**Table 9:** Top gene RNA-sequencing-based expression in human donor macular retinal or RPE tissue isolated using the Utah Protocol:

hg19 Position	Gene Name	Tissue Type with Higher Expression	Fold Change between RPE and Retina	P-value
chr16:89985572–90002500	TIJBB3	Neurosensory Retma	35.201	1.03E-130
chr8:99037078–99058697	RPL30	RPE	2.974	5.69259E-61
chr13:36345477–36705443	DCLK1	Neurosensory Retina	10.303	6.27566E-29
chr7:1031 12230–103629963	RELN	Neurosensory Retina	10.599	2,41595E-28
chr7:21467651–21554440	SP4	Neurosensory Retina	2.467	2,09322E-23
chr16:90014332–9003446S	DEFS	Neurosensory Retina	1.709	1.10S65E-20
chr7:90013034–90142716	CLDN12	Neurosensory Retina	2.050	7.47808E-16
chr7:42000547–42277469	GL13	RPE	2.454	3.30197E-15
chr9:B314245–10612723	PTPRD	Neurosensory Retina	3.962	5.21447E-13
chr2:162848750–162931052	DPP4	RPE	2.725	2.96267E-OS
chr13:111766158–111768025	ARIIGEF7	Neurosensory Retina	2.276	3,91738E-05

Differential expression between macular retinal and RPE/Choroid was calculated and p-values corrected for multiple testing using Benjamini-Hockberg. The absolute fold change is represented between the tissue with higher expression versus the tissue with lower expression.
